# *Mycobacterium leprae* alters classical activation of human monocytes in vitro

**DOI:** 10.1186/s12950-016-0117-4

**Published:** 2016-03-11

**Authors:** Dorothy Fallows, Blas Peixoto, Gilla Kaplan, Claudia Manca

**Affiliations:** Public Health Research Institute, New Jersey Medical School, Rutgers University, Newark, NJ USA; The Bill & Melinda Gates Foundation, Seattle, WA USA

**Keywords:** *Mycobacterium leprae*, Polarization, BCG vaccination, Cytokine, Macrophage, Innate immunity, Leprosy

## Abstract

**Background:**

Macrophages play a central role in the pathogenesis of leprosy, caused by *Mycobacterium leprae.* The polarized clinical presentations in leprosy are associated with differential immune activation. In tuberculoid leprosy, macrophages show a classical activation phenotype (M1), while macrophages in lepromatous disease display characteristics of alternative activation (M2). Bacille Calmette-Guérin (BCG) vaccination, which protects against leprosy, can promote sustained changes in monocyte response to unrelated pathogens and may preferentially direct monocytes towards an M1 protective phenotype. We previously reported that *M. leprae* can dampen the response of naïve human monocytes to a strong inducer of pro-inflammatory cytokines, such as BCG. Here, we investigated the ability of the pathogen to alter the direction of macrophage polarization and the impact of BCG vaccination on the monocyte response to *M. leprae*.

**Findings:**

We show that in vitro exposure of monocytes from healthy donors to *M. leprae* interferes with subsequent M1 polarization, indicated by lower levels of M1-associated cytokine/chemokines released and reduced expression of M1 cell surface markers. Exposure to *M. leprae* phenolic glycolipid (PGL) 1, instead of whole bacteria, demonstrated a similar effect on M1 cytokine/chemokine release. In addition, we found that monocytes from 10-week old BCG-vaccinated infants released higher levels of the pro-inflammatory cytokines TNF-α and IL-1β in response to *M. leprae* compared to those from unvaccinated infants.

**Conclusion:**

Exposure to *M. leprae* has an inhibitory effect on M1 macrophage polarization, likely mediated through PGL-1. By directing monocyte/macrophages preferentially towards M1 activation, BCG vaccination may render the cells more refractory to the inhibitory effects of subsequent *M. leprae* infection.

## Background

Macrophages play a central role in the pathogenesis of leprosy, a chronic debilitating disease caused by *Mycobacterium leprae*. Monocyte/macrophages show a dynamic plasticity, allowing them to respond to environmental stimuli by presenting a classical (M1) or alternative (M2) activation state [1]. M1-activated macrophages release high levels of pro-inflammatory cytokines with enhanced microbicidal activity; M2 macrophages produce inhibitory cytokines and are less responsive to stimuli [2, 3]. Leprosy presents as a spectrum of clinical manifestations, associated with differential immune activation. While tuberculoid leprosy shows robust cell-mediated immunity with predominantly M1-activated macrophages, lepromatous disease is characterized by strong humoral immunity and macrophages show an M2 phenotype [4, 5].

Bacille Calmette-Guérin (BCG) vaccination protects against leprosy and is associated with reduced burden of unrelated diseases, suggesting non-specific protection that may involve shaping innate immunity [6–8]. BCG vaccination has been shown to induce sustained changes in the phenotype of circulating monocytes, with greater pro-inflammatory cytokine production [9]. Moreover, ex vivo stimulation of peripheral blood mononuclear cells (PBMC) from 10-week old infants vaccinated at birth with BCG, revealed a gene expression signature similar to an M1 macrophage profile with down-regulation of M2-associated genes [10].

We previously reported that stimulation of naïve monocytes from healthy donors with *M. leprae* alone, or *M. leprae* followed by BCG, induced the release of cytokine/chemokines that are associated with negative regulation of inflammation [11]. *M. leprae* itself was a poor inducer of the pro-inflammatory cytokine TNF-α, consistent with other reports [12]. However, when naïve monocytes were first stimulated by BCG and then exposed to *M. leprae*, the cells produced a pro-inflammatory cytokine profile matching that of BCG alone [11]. Here, we investigated the ability of *M. leprae* to interfere with M1 maturation of monocyte induced by exposure to IFN-γ and M2 maturation induced by exposure to IL4/IL13 in vitro. We also tested whether BCG vaccination, by favoring an M1 phenotype, may render the cells resistant to the inhibitory effects of *M. leprae*.

## Methods

### Reagents

*Mycobacterium leprae* Thai-53 from the National Hansen’s Disease Programs Laboratory Research, Louisiana State University, Baton Rouge (American Leprosy Missions and the Society of St. Lazarus) [13–15] and BCG from Trudeau Institute (Mycobacterial Culture Collection No. 1011) were prepared as described [11]. Purified *M. leprae* PGL-1, obtained through the NIH Biodefense and Emerging Infections Research Resources Repository NIAID, NIH: NR-19342, was used as described [16]. Peripheral blood mononuclear cells (PBMCs) from 10-week old infants unvaccinated (*N* = 18) or BCG-vaccinated at birth (*N* = 20) were provided by Dr. Willem Hanekom, University of Cape Town, South Africa.

These studies were approved by the Institutional Review Boards of Rutgers University and the University of Cape Town (Pro2012001418, Pro0120110233).

### Monocyte preparation/stimulation

PBMCs were isolated from buffy coat (New Jersey Blood Center) of healthy donors; monocytes were purified using anti-CD14 antibody-conjugated magnetic beads (Miltenyi Biotec, Auburn, CA) and allowed to adhere before treatment [11]. For M1 polarization, monocytes were exposed to IFN-γ (10 ng/ml) for 24 h, then stimulated with LPS (100 ng/ml) for 19 h [3, 17]. M2 polarization was achieved by treatment with IL-4/IL-13(10 ng/ml each) for 24 h [18, 19]. To determine the impact of *M. leprae*, cells were exposed to *M. leprae* at multiplicity of infection (MOI) 5:1 or 20:1 (bacilli:cells) for 5 h prior to IFN-γ priming (M1) or IL-4/IL-13 treatment (M2). Cell viability was confirmed by trypan blue exclusion (Life Technologies, CA). Controls included: M1 or M2 polarization alone, *M. leprae*-stimulation alone, and unstimulated/untreated cells.

Monocytes were isolated from infant PBMCs by adherence [11], plated in 96-well plates (1x10^5^ cells/well) in RPMI 1640/20 % human serum, and exposed to *M. leprae* (MOI 5:1) or culture medium alone.

### Analysis

Cell supernatants were removed after 48 h (M1) or 24 h (M2) and probed using a multiplex human cytokine/chemokine panel (Bio-Rad, Hercules, CA) according to manufacturer’s instructions. Data/statistical analysis were done as described [16]. Cells were detached with EDTA (20 mM in PBS), washed with PBS, incubated with fluorescently-labeled antibodies against: CCR7, HLA-DR, CD80, CD86, and CD40 (BD Bioscience) (M1) or CD23 (M2), and analyzed on a FACSCalibur (BD Biosciences) using CellQuest (BD Biosciences) and FlowJo (Tree Star) software. Greater than 2,000,000 events per sample were analyzed.

## Results and Discussion

**Fig. 1:**
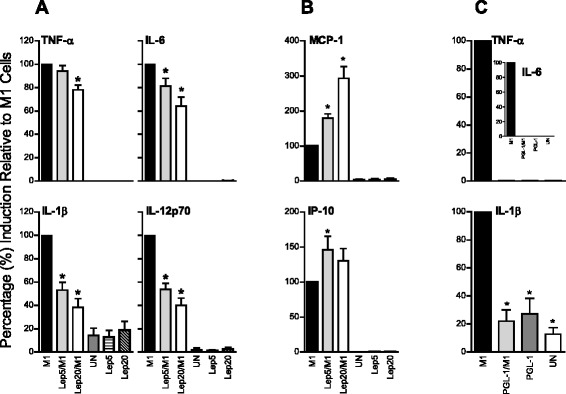
*M. leprae* and its lipid component PGL-1 alter the release of cytokines and chemokines from M1 polarized monocytes. CD14^+^ cells were polarized towards M1 phenotype (M1) or pre-exposed for 5 h to the following: **a**-**b** *M. leprae* (MOI 5:1, Lep5 or 20:1, Lep20), **c** PGL-1 (50 μg/mL) or cultured in medium alone (UN). Cell supernatants were collected at 48 h and cytokines/chemokines analyzed. Results are the mean ±  Sem: **a**-**b** 8 experiments (8 independent donors); and **c** 6 experiments (6 independent donors) performed in duplicate. A 2-tailed paired *t*-test was used for statistical analysis (*P ≤ 0.05, relative to M1 cells)

M1 polarized monocytes (positive control) released high levels of TNF-α (mean 123.4 ± 17.0 ng/ml), IL-6 (152.1 ± 175.3 ng/ml), IL-1β (0.3 ± 0.07 ng/ml), IL-12p70 (2.1 ± 0.8 ng/ml), MCP-1 (6.2 ± 2 ng/ml), IP-10 (121 ± 18.9), Rantes (11.6 ± 2.3 ng/ml), MIP-1α (22.5 ± 3.2 ng/ml) and MIP-1β (88.7 ± 26.7 ng/ml) compared to unstimulated/untreated cells. Exposure of cells to *M. leprae* (MOI 5:1) prior to M1 polarization significantly reduced levels of IL-6, IL-1β and IL-12p70 by 19 % ± 27, 47 % ± 25, and 46 % ± 20, respectively, compared to M1 controls (Fig. 1a). Pre-stimulation with *M. leprae* at higher MOI (20:1) led to further reduction; IL-6, IL-1β and IL-12p70 were reduced by 36 % ± 32, 62 % ± 28 and 60 % ± 25, respectively. TNF-α was also significantly lowered by 22.2 % ±5.0 in response to *M. leprae* pre-stimulation, but only at higher MOI. In contrast, MCP-1 and IP-10 levels were significantly increased (*P* ≤ 0.0001 and *P* ≤ 0.05, respectively) by *M. leprae* (MOI 5:1) pre-stimulation compared to M1 controls (Fig. 1b). While *M. leprae* at the higher MOI further increased levels of MCP-1 (*P* ≤ 0.001), IP-10 levels were not significantly different at the two MOIs (Fig. 1b). No consistent differences were seen in Rantes, MIP-1α and MIP-1β (data not shown), suggesting that the impact of *M. leprae* on M1 polarization was selective. The inhibitory effect of *M. leprae* on M1 polarization observed here may involve interference with IFN-γ signaling, as described in monocyte/macrophages exposed to *Mycobacterium tuberculosis* and in *M. leprae*-infected nude mice [20–22]. When monocytes were pre-exposed to PGL-1, instead of whole bacteria, the results were even more dramatic (Fig. 1c). M1 polarized controls released high levels of TNF-α (mean 24,243 ± 7,182 pg/ml), IL-6 (50,348 ± 8913 pg/ml) and IL-1β (75.5 ± 23.2 pg/ml), which were significantly reduced by PGL-1 pre-exposure (TNF-α: 6.7 ± 3.9 pg/ml; IL-6:18.3 ± 3.9 pg/ml; IL-1β:12.6 ± 2.0 pg/ml). PGL-1 alone induced cytokine levels comparable to unstimulated/untreated controls. These results support previous reports that PGL-1 is an important determinant in *M. leprae*-monocyte interactions [16, 23, 24].

**Fig. 2:**
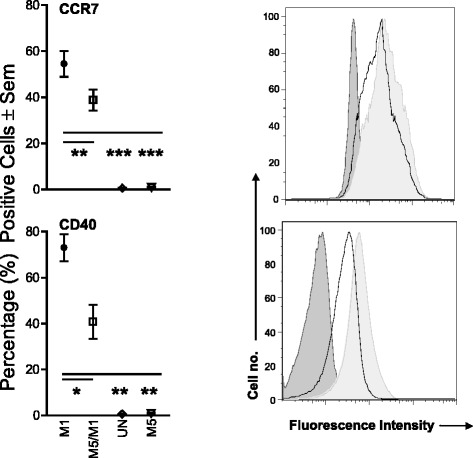
Effect *of M. leprae* on M1 surface marker expression. CD14^+^ cells were seeded in 24-well plates at the concentration of 5x10^5^/well and polarized (M1), or exposed to *M. leprae* at MOI 5:1 for 5 h prior to polarization (Lep5/M1). Additional conditions included stimulation of monocytes with *M. leprae* at MOI 5:1 (Lep5) and cells in culture medium alone without stimulation (UN). Changes in the percentages of CCR7^+^ and CD40^+^ cells were evaluated. A two-tail paired Student’s *t*-test was used for the analysis. Results are representative of 6 experiments (6 individual donors); Histogram of one representative experiment. Dark gray: unstimulated cells; light gray: M1 cells; black line: Lep5/M1. *P ≤ 0.05; **P ≤ 0.001; ***P ≤ 0.0001

M1 polarization also resulted in significantly increased percentages of cells expressing the surface markers CCR7 and CD40, relative to unstimulated/untreated controls. When monocytes were pre-exposed to *M. leprae* at low MOI, the percentages of CCR7^+^ (*P* ≤ 0.001) and CD40^+^ (*P* ≤ 0.05) cells were reduced compared to M1 polarization alone (Fig. 2). Pre-stimulation with *M. leprae* at higher MOI did not result in further reduction (data not shown). HLA-DR^+^, CD80^+^ and CD86^+^ M1 cell percentages were unaffected by *M. leprae* pre-stimulation. The mean fluorescence intensities (MFI) of CCR7, CD40 and CD80 in M1 cells were also significantly reduced by *M. leprae* pre-exposure (Table 1). *M. leprae* alone was comparable to unstimulated/untreated controls.

**Table 1:** *M. leprae* effect on the MFI of M1 surface markers

	Phenotypic markers		
	CCR7	CD40	CD80
M1 cells	248.0 ± 31.7	51.6 ± 6.3	765.0 ± 64.2
Lep5 (5 h)/M1	167.1** ± 22.6	40.4* ± 6.5	681.9 ± 77.6
Lep20 (5 h)/M1	152.2* ± 7.9	46.3 ± 7.8	591.0* ± 70.1
Unstimulated cells	44.3** ± 1.7	6.5*** ± 0.3	16.6*** ± 2.8
Lep5	48.4** ± 1.6	7.8*** ± 0.3	20.3*** ± 2.8
Lep20	49.3** ± 1.4	7.8** ± 0.4	25.3*** ± 3.3

The values represent the mean MFI ± Sem of 4–7 independent experiments (independent donors) for each condition. The statistical significance is shown as compared to the M1 cells. Lep5 (*M. leprae* MOI 5:1); Lep20 (*M. leprae* MOI 20:1).*P ≤ 0.05; **P ≤ 0.001 and ***P ≤ 0.0001

In contrast, the impact of *M. leprae* on expression of M2 macrophage markers was minimal. Pre-stimulation with *M. leprae* increased the MFI of CD23 (295 ± 116 and 388.5 ± 153 at MOI 5:1 and 20:1, respectively) over M2 polarization alone (242.7 ± 108), with low levels produced by unstimulated/untreated cells (24.9 ± 12.3), and had no effect on IL-1Ra and IL-10 (data not shown). Thus, the effect of *M. leprae* on monocytes is primarily due to inhibition of M1 activation and does not appear to significantly affect M2 polarization.

**Fig. 3:**
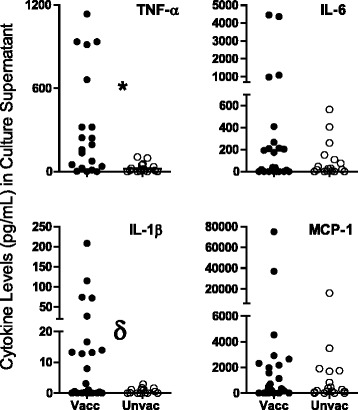
Differential TNF-α and IL-1β response to *M. leprae* stimulation of monocytes isolated from 10-weeks old BCG-vaccinated and unvaccinated infants. Monocytes were isolated from PBMCs of 10-week old infants, unvaccinated or BCG-vaccinated at birth, and stimulated for 24 h with *M. leprae*. Data are expressed in pg/ml minus the value of the corresponding unstimulated controls. A 2-tailed paired *t*-test was used for statistical analysis between cells stimulated with *M. leprae*. *P ≤ 0.001; ^δ^ *P* ≤ 0.05

Finally, we compared the effect of *M. leprae* on monocytes from 10-week old unvaccinated or BCG-vaccinated infants (Fig. 3). Levels of TNF-α and IL-1β released in response to *M. leprae* were significantly higher in monocytes from vaccinated infants than those from unvaccinated infants, while IL-6 and MCP-1 showed trends towards higher levels in vaccinated versus unvaccinated infants. These results demonstrate that in vivo activation of monocytes due to BCG vaccination may render the cells refractory to the inhibitory effects of *M. leprae*.

## Conclusion

Exposure to *M. leprae* can alter the functional capacity of monocytes, which may diminish the efficacy of the host response to subsequent stimuli. Our results support increasing evidence suggesting that the innate immune response may be shaped by prior history of exposure, which could also explain the protection afforded by BCG vaccination against *M. leprae* and other unrelated pathogens.

## Abbreviations

BCG: Bacille Calmette-Guérin
PGL-1: Phenolic glycolipid 1
PBMC: Peripheral blood mononuclear cells
M1: Classical macrophage activation
M2: Alternative macrophage activation
TNF-α: Tumor necrosis factor alpha
IL-1β: Interleukin 1 beta
IL-12p70: Interleukin 12p70
IL-6: Interleukin 6
MCP-1: Monocyte chemotactic protein 1
IP-10: Interferon gamma-inducible protein 10
Rantes: Regulated on activation, normal T cell expressed and secreted
MIP-1α: Macrophage inflammatory protein 1 alpha
MIP-1β: Macrophage inflammatory protein 1 beta
IFN-γ: Interferon gamma
MOI: Multiplicity of infection
CCR7: C-C chemokine receptor type 7
HLA-DR: Human leukocyte antigen - antigen D related
CD40: Cluster of differentiation 40
CD80: Cluster of differentiation 80
